# External quality assessment for arbovirus diagnostics in the World Health Organization Western Pacific Region, 2013–2016: improving laboratory quality over the years

**DOI:** 10.5365/wpsar.2017.8.3.001

**Published:** 2017-09-29

**Authors:** Mohammad Yazid Abdad, Raynal C Squires, Sebastien Cognat, Christopher John Oxenford, Frank Konings

**Affiliations:** a Health Emergencies Programme, World Health Organization Regional Office for the Western Pacific, Manila, Philippines.; b Health Emergencies Programme, World Health Organization, Lyon, France.

## Abstract

Arboviruses continue to pose serious public health threats in the World Health Organization (WHO) Western Pacific Region. As such, laboratories need to be equipped for their accurate detection. In 2011, to ensure test proficiency, the WHO Regional Office for the Western Pacific piloted an external quality assessment (EQA) programme for arbovirus diagnostics. By 2016, it had grown into a global programme with participation of 96 laboratories worldwide, including 25 laboratories from 19 countries, territories and areas in the Region. The test performance of the 25 laboratories in the Region in 2016 was high with 23 (92%) reporting correct results in all specimens for dengue and chikungunya viruses. For Zika virus, 18 (72%) of the 25 laboratories reported correct results in all specimens, while seven (28%) demonstrated at least one error. When comparing iterations of this EQA programme in the Region between 2013 and 2016, the number of participating laboratories increased from 18 to 25. The first round only included dengue virus, while the latest round additionally included chikungunya, Zika and yellow fever viruses. Proficiency for molecular detection of dengue virus remained high (83–94%) over the four-year period. The observed proficiency for arbovirus diagnostics between 2013 and 2016 is an indicator of laboratory quality improvement in the Region.

Arboviruses continue to pose a serious threat to human health worldwide as evidenced by the emergence and re-emergence of arboviral infections in the form of outbreaks globally in the last five years. During this time, the world witnessed the re-emergence of chikungunya virus (CHIKV) and Zika virus (ZIKV) while dengue virus (DENV) and yellow fever virus (YFV) continued to circulate widely. In February 2016, the Director-General of the World Health Organization (WHO) declared a Public Health Emergency of International Concern (PHEIC) in response to ZIKV, microcephaly and other neurological disorders reported in Brazil, following a similar cluster in French Polynesia in 2014. (*1*) This declaration ended in November 2016 in favour of a longer-term programmatic approach. (*2*)

The WHO Western Pacific Region bears a large arboviral disease burden. For dengue alone, there were more than 860 000 cases and 1500 deaths reported between 2013 and 2016 (WHO Regional Office for the Western Pacific, unpublished data, 2017). CHIKV is endemic in the Region and considered a threat to the Pacific island countries and areas with recent outbreaks in New Caledonia and Papua New Guinea. (*3*) The first recorded outbreak of ZIKV disease in the Region was in the Federated States of Micronesia in 2007. (*4*) The virus has since been detected in the majority of the countries and occasionally causes outbreaks such as in Singapore in 2016. (*5*)

Preparedness in the face of such potential disease occurrences is paramount. Therefore, public health laboratories and their equivalent in the research sector throughout the Region have developed or adopted molecular methods for arbovirus detection. These new assays complement serological assays already in use and allow for diagnosis in the early stages of illness. To assess the competency of laboratories in the Region, an external quality assessment (EQA) programme for arbovirus diagnostics was developed in 2011 with the goal of enabling the laboratories to gauge their proficiency and to identify areas for improvement. While test capacity is an indication of whether laboratories have the necessary elements, including required equipment, reagents and protocols in place to perform a test and to gauge the throughput, proficiency relates to how reliably and accurately a test is performed. The establishment of an EQA programme for dengue diagnostics was part of the *Asia Pacific Strategy for Emerging Diseases (2010)*, an action framework for building International Health Regulations core capacities. (*6*) Participation in EQA programmes is a requirement for achieving the International Organization for Standardization 15189 accreditation, which specifies the quality management system requirements particular to medical laboratories. (*7*)

The development of the EQA programme involved the main reference laboratories and collaborating centres in the Region (**Fig. 1**). A pilot to assess the feasibility, including panel preparation and logistics, of EQA for dengue diagnostics was first conducted by the National Institute of Infectious Diseases, Japan in 2011. This informed the development of the first round of EQA for dengue, which was introduced in 2013. (*8*) Eighteen laboratories participated in the programme, which was coordinated by the Environmental Health Institute (EHI), Singapore, a WHO Collaborating Centre for Reference and Research of Arbovirus and their Associated Vectors. There was no round of EQA in 2014 due to logistic and technical review of the first round for improvement of subsequent iterations. In 2015, a second round of EQA, not only for dengue but also chikungunya diagnostics, was prepared by EHI (*9*) and involved 24 laboratories, including 19 in the Region and five in the WHO South-East Asia Region. In 2016, in succession to the two regional programmes, WHO organized the first global EQA programme for arbovirus diagnostics. The programme was developed and managed by the Royal College of Pathologists of Australasia Quality Assurance Programs. As of July 2017, global participation stands at 96 laboratories throughout all WHO regions.

**Fig. 1:**
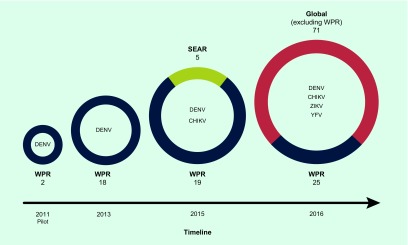
Increase in the number of participating laboratories, geographic coverage and variety of pathogens of the WHO EQA programme for arbovirus diagnostics from pilot to global programme, 2011–2016

Legend: Evolution of the EQA programme for arbovirus diagnostics from 2011 to 2016 showing an increase in number of participating laboratories, geographic coverage and variety of pathogens included. No round of EQA was conducted in 2014. Circles and their relative size provide an illustration of this growth over time and by WHO region. Numbers under respective region or global indicate number of participating laboratories. Pathogens included in each panel are listed inside each circle. CHIKV = chikungunya virus; DENV = dengue virus; Global (red); SEAR = South-East Asia Region (green); WPR = Western Pacific Region (dark blue); YFV = yellow fever virus; and ZIKV = Zika virus.

In 2016, 26 laboratories in the Region were invited to participate in the EQA programme. This iteration of the EQA assessed participating laboratories’ capacity and proficiency for DENV, CHIKV, ZIKV and YFV (optional) diagnosis by polymerase chain reaction (PCR). Panels containing blinded samples of various dilutions of the four arboviruses to be identified by participating laboratories were shipped between November and December 2016. Of the 26 laboratories invited, 25 laboratories from 19 countries, territories and areas in the Region returned results; one laboratory was unable to participate due to logistical issues preventing delivery of the panel. All 25 laboratories participated in PCR diagnosis of the three required viruses; additionally, 22 performed DENV serotyping and 13 participated in the optional YFV component of the EQA. Results from the 2016 EQA are presented in **Fig. 2**.

**Fig. 2:**
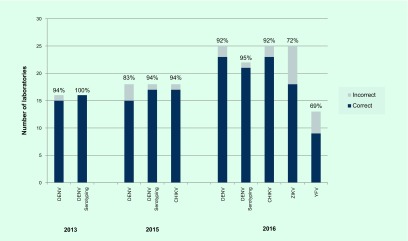
Proficiency* of laboratories in the WHO Western Pacific Region participating in the EQA programme for arbovirus diagnostics, 2013–2016

Legend: CHIKV = chikungunya virus; DENV = dengue virus; YFV = yellow fever virus; and ZIKV = Zika virus.

*Proficiency is defined as the percentage of laboratories reporting all correct results for a component of the EQA panel. No round of EQA was conducted in 2014.

In 2016, test performance was high with 23 of 25 (92%) laboratories reporting correct results in all specimens in the panel for DENV and CHIKV. For ZIKV, 18 (72%) laboratories reported correct results in all specimens, while seven (28%) demonstrated at least one error. Twenty-one of 22 (95%) laboratories were able to correctly identify DENV serotypes. All four laboratories that performed ZIKV lineage testing correctly determined whether the strain was of Asian or African lineage. Overall, errors appeared to be randomly distributed, and no patterns of inaccuracy could be observed for particular specimens or laboratories (data not shown). Of the 13 laboratories that took part in the optional module for detection of YFV, nine (69%) successfully identified YFV in test specimens. While not endemic or reported in Asia, demonstrating regional preparedness to detect and confirm YFV is vital in the event an outbreak were to occur. The importance of having diagnostic capacity for YFV was highlighted by the recent importations of laboratory-confirmed YFV cases from Angola into China. (*10*)

A laboratory preparedness survey to determine test capacity for arboviruses in the Region was conducted in February 2016, immediately after the declaration of a PHEIC related to ZIKV. (*11*) It revealed that of the 19 national-level public health laboratories surveyed, 16 (84%) reported molecular testing capacity for ZIKV. The results of the 2016 EQA for arboviruses indicate that, in addition to test capacity, laboratories have a high level of test proficiency for ZIKV. Additionally, three countries that indicated not having test capacity at the time of the survey had this capacity in place at the time of the 2016 EQA (data not shown).

When comparing the results of the EQA programmes in the Region between 2013 and 2016, EQA participation has consistently increased since 2013 (**Fig. 2**). Laboratories in the Region demonstrated good proficiency at detecting DENV in 2013 and both DENV and CHIKV in 2015 with more than 83% of laboratories reporting correct results for all specimens in the panels. The Region can now claim at least 23 national-level public health laboratories with consistently high accurate molecular detection capacity for DENV and CHIKV compared to previous years. The observed increase in participation and improvements in EQA results suggest that laboratory managers are continuously improving their laboratory operations. Their commitment is important in making sure that the Region is prepared for health emergencies, supporting routine surveillance and providing accurate diagnoses.

The EQA programme is an evolving tool that will continue to be reviewed and changed to monitor and inform improvement of laboratory diagnostic testing for arboviral diseases. It shows that small initiatives can grow into larger accomplishments in a stepwise manner when sufficient investments are made and when the impact on public health by better surveillance and response is recognized. The observation of an increasing number of participants and high proficiency between 2013 and 2016 is an indicator of improvement in laboratory capacity and performance in the Region.
